# Low levels of Vitamin D during pregnancy associated with gestational diabetes mellitus and low birth weight: results from the MAASTHI birth cohort

**DOI:** 10.3389/fnut.2024.1352617

**Published:** 2024-06-03

**Authors:** R. Deepa, Onno C. P. Van Schayck, Giridhara R. Babu

**Affiliations:** ^1^Indian Institute of Public Health—Bengaluru, Public Health Foundation of India, Bengaluru, India; ^2^Department of Family Medicine, Care and Public Health Research Institute, Maastricht University, Maastricht, Netherlands; ^3^Department of Population Medicine, College of Medicine, QU Health, Qatar University, Doha, Qatar

**Keywords:** vitamin (25[OH]D), gestational (gestational diabetes), low birth weight, low- and middle-income countries (LMIC), insulin resistance

## Abstract

**Introduction:**

India has a high prevalence of Vitamin D insufficiency among women of childbearing age. In this study, we aimed to evaluate the potential relationship between Vitamin D deficiency and gestational diabetes mellitus (GDM) and low birth weight (LBW) of newborns in the “Maternal antecedents of adiposity and studying the transgenerational role of hyperglycaemia and insulin” (MAASTHI) birth cohort.

**Methods:**

A prospective cohort study involving 230 participants was conducted in public hospitals located in urban Bengaluru, India. Healthy pregnant women who visited these hospitals for antenatal care (ANC) and who were between 14 and 36 weeks of gestational age were recruited after obtaining their informed consent. An oral glucose tolerance test (OGTT) was administered between 24 and 36 weeks of pregnancy and blood samples were preserved at −80°C for Vitamin D analysis. Follow-up at birth included recording the child's birth weight.

**Results:**

We found that 178 (77.4%) of the study participants were vitamin D deficient, 44 (19.1%) were diagnosed with GDM, and 64 (27.8%) gave birth to LBW babies. Women in the lowest quartile of serum Vitamin D levels had three times higher odds of developing GDM than women in the higher quartiles [OR = 3.22 (95% CI: 1.03, 10.07), *p* = 0.04] after adjusting for age, parity, socioeconomic status, season, and adiposity. For every one-unit increase in Vitamin D levels, Homeostatic Model Assessment for Insulin Resistance (HOMA-IR) decreased by nearly 18%. Furthermore, causal mediation analysis showed that a decrease in one unit of Vitamin D is associated with a decrease of 0.015 units of fasting blood sugar (FBS) and 0.019 units of postprandial blood sugar (PPBS) as it flows through the mediator variable insulin resistance. Vitamin D-deficient women were twice at risk of giving birth to LBW babies (OR 2.04, 95% CI 0.99, 4.19, *p* = 0.05).

**Discussions:**

Low levels of Vitamin D during pregnancy are associated with a greater risk of pregnant women developing GDM and giving birth to LBW babies in urban Bengaluru.

## Introduction

Vitamin D deficiency is common among pregnant women worldwide due to fetal growth, low exposure to sunlight, and low dietary intake. Studies have shown the highest prevalence of Vitamin D deficiency in Asia (1). Despite being a tropical nation with plenty of sunshine, India has a high prevalence of Vitamin D insufficiency among women of childbearing age. A recent review estimated a pooled prevalence of 32.35% of Vitamin D deficiency among healthy pregnant women (2). Evidence from South India shows that 60–80% of mothers were Vitamin D insufficient and more than 30% were severely Vitamin D deficient (3, 4). During pregnancy, there is significant emphasis on the significance of serum 25-hydroxyvitamin D (25(OH)D) because the fetus relies entirely on the mother as its source of 25(OH)D. 25(OH)D is the major circulating metabolite of Vitamin D and is currently regarded as the most effective measure of the body's Vitamin D availability, originating from both endogenous synthesis and exogenous intake. Vitamin D is capable of crossing the placenta, facilitated in part by the megalin–cubilin endocytic mechanism (5). Studies have shown that adverse health outcomes due to insufficiency in Vitamin D level leads to gestational diabetes, preeclampsia, preterm birth, low birth weight, and intrauterine death (6–9).

The Vitamin D receptor is expressed in a variety of cell types throughout the body. Vitamin D may play a role in insulin response and other cellular functions. Increasing evidence suggests that Vitamin D could be pivotal in maintaining normal glucose homeostasis. *In vivo*, Vitamin D deficiency causes dysregulation of glucose metabolism by increasing insulin resistance through deteriorating β-cell function and mass. During pregnancy, the disruption of normal glucose homeostasis causes insulin resistance, resulting in fetal macrosomia and increased adiposity in infants (10, 11). A decreased amount of serum 25(OH)D, calcitriol [1,25(OH)2D], and raised parathyroid hormone (PTH) can increase intracellular calcium in adipocytes, which can stimulate lipogenesis, predisposing the individual to further weight gain and thus increasing the risk of diabetes (12). Vitamin D insufficiency and the occurrence of insulin resistance have been reported in several studies (13, 14). 25(OH)D was inversely and independently associated with insulin resistance in only those women who were Vitamin D-deficient (15).

Vitamin D plays a crucial role in the development of the fetus through its interaction with parathyroid hormone and the regulation of calcium levels. Research has shown that inadequate levels of Vitamin D during both pregnancy and early life can have a significant impact on proper bone mineralization in women, which is strongly associated with them giving birth to infants with low birth weight. Animal studies have shown that deficiency in Vitamin D causes placental insufficiency and fetal intrauterine growth retardation by inducing placental inflammation in mice (16). It is important to note that there is a positive connection between maternal Vitamin D levels during pregnancy and the levels of Vitamin D in the newborn's blood (17). This finding suggests that insufficient maternal Vitamin D levels could also potentially have a negative effect on child development.

This study aimed to examine the relationship between maternal Vitamin D status and gestational diabetes while exploring the mediation role of insulin resistance among a cohort of healthy pregnant women attending public hospitals in Bengaluru. We also aimed to look at the impact of Vitamin D on other pregnancy-related complications like hypertension, preterm birth, and low birth weight.

## Materials and methods

### Design

**Figure 1:**
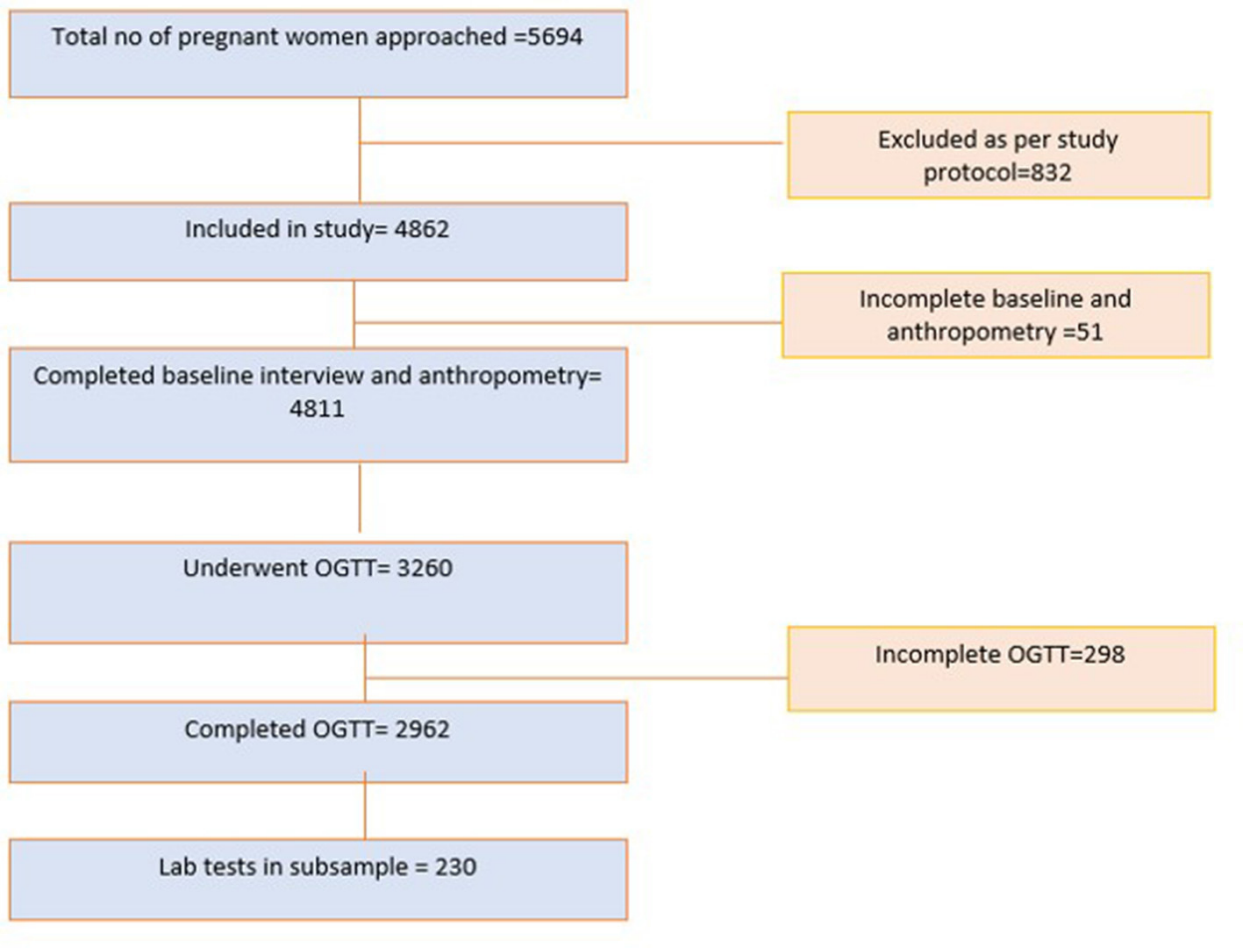
Flowchart of the recruitment in MAASTHI and final sample included in the analysis.

The study is nested within an ongoing study, the “Maternal antecedents of adiposity and studying the transgenerational role of hyperglycaemia and insulin” (MAASTHI), a prospective cohort of healthy pregnant women in urban primary healthcare centers and referral hospitals of Bengaluru. MAASTHI was set up to understand the effects of maternal physiological, nutritional, and psychosocial environment on the risk factors of non-communicable diseases in the child. Pregnant women were recruited between 2016 to 2018. The eligibility criteria comprised women older than 18 years and those who were delivering at the study site. Women with diabetes, HIV, and hepatitis infection were excluded from the study. Women and their newborns were followed up soon after delivery. Approximately 4,862 pregnant women in their 14–36 weeks of pregnancy were recruited from public hospitals, of which 4,811 completed the baseline questionnaire and 2,962 women completed oral glucose tolerance test (OGTT) between 24 and 36 weeks. Their blood samples were stored in a biorepository for future micronutrient tests. At birth, follow-up was completed within 72 h after delivery; 863 women had completed delivery follow-up at the time of analysis. Vitamin D, insulin, and other micronutrients were analyzed in 2019–20 (Figure 1).

The sample size for studying the association between Vitamin D was estimated based on previous studies where the prevalence of GDM was 14%, with 80% power and 95% confidence interval using 10% precision. The sample size obtained was 91. After accounting for a 60% non-response rate, the final sample size obtained was 228; hence, 230 samples were randomly chosen from the MAASTHI cohort to perform the micronutrient assessment.

### Data collection

Sociodemographic details, obstetric history, family history of non-communicable disease (NCD), food habits, 24-h diet recall, and physical activity were recorded using an interviewer-administered questionnaire from pregnant women who were between 14 and 36 weeks in their pregnancy during the baseline visit. The food habits of the participants were recorded to elicit the frequency of consumption of food groups like dairy, meat, and seafood in the last month. We recorded their blood pressure, height, weight, and skin-fold thickness. Data were collected only once during pregnancy except 24-h recall, which was administered twice during pregnancy.

OGTT was conducted between 24 and 36 weeks of pregnancy. For this test, 2 ml blood was collected twice in Sodium Fluoride (NaF) vacutainers for estimating fasting and 2-h postprandial glucose levels after ingesting 75 g of glucose. For hemoglobin assessment, 3 ml of blood was collected in an EDTA vacutainer.

A 6-ml blood sample was collected in a plain vacutainer from each participant by a trained phlebotomist using the venepuncture procedure. The vacutainer was wrapped with aluminum foil to protect it from sunlight and kept for 45 min to coagulate. The sample was then centrifuged for 10 min at 1,500 rpm/min. A clear supernatant serum was transferred into black color cryovials. Serum samples were stored at −80°C in a biorepository. In the study, 230 respondents were randomly chosen for Vitamin D and insulin analysis using chemiluminescent microparticle immunoassay (CMIA) in Abbott ARCHITECT i2000/i2000SR. ARCHITECT 25-OH Vitamin D 5P02 reagents were used for this test. We combined sample, assay diluent, and paramagnetic anti-vitamin D-coated microparticles. 25-OH vitamin D present in the sample is displaced from the Vitamin D binding protein and binds to anti-vitamin D-coated microparticles, forming an antigen–antibody complex. After serum incubation, a conjugate containing acridinium-labeled Vitamin D is added to the reaction mixture and binds to unoccupied binding sites of the anti-vitamin D-coated microparticles. After further incubation and washing, pre-trigger and trigger solutions are added to the reaction mixture. The resulting chemiluminescent reaction is measured as relative light units (RLUs). There is a relationship between the amount of 25-OH vitamin D in the sample and the RLUs detected by the ARCHITECT iSystem Optics. The results are calculated automatically based on the previously established calibration curve.

All samples were tested in the same run at the end of the study. Internal and external quality checks were performed; immunoassay premium plus tri-level controls were used. The External Quality Assurance Scheme (EQAS) coefficient of variation (%) for our method of Vitamin D analysis was 17.8.

Newborn anthropometry was performed using SECA 354 Weighing Scale and SECA 417 infantometer. We measured the mid-upper arm circumference (MUAC) using Chasmors body circumference tape. The sum of biceps, triceps, and subscapular (SFT) was measured on the left side of the body using the Holtain Calipers (Holtain, UK). The research staff were trained and certified annually for anthropometry assessment from St. John's Research Institute, Bengaluru. Two readings of weight in kilograms were taken to the nearest 0.5 g, length, and MUAC was measured in centimeters, and skin-fold thickness in millimeters to the nearest 0.2 mm.

### Quantitative variables

Socioeconomic status was assessed using the Kuppuswamy socioeconomic scale, which considers education, occupation, and income. The status was classified into five categories: upper, upper middle, lower middle, upper lower, and lower. For analysis, these categories were condensed into two main groups: lower (including upper lower and lower) and middle (including upper middle and lower middle)—were created (18). The physical activity questionnaire during pregnancy was administered at recruitment. The questionnaire covered various aspects of physical activity, including exercise, hobbies, household chores, sedentary activities, and daily routines. For each activity, we noted the frequency per week and the duration in minutes. To determine the overall intensity of physical activity, we calculated the metabolic equivalent (MET) values for each activity, considering the assigned MET value, activity duration, and weekly frequency. By summing up the MET values for all individual physical activities, we arrived at the combined MET value. Hypertension among participants was categorized into normal (<120/80 mmHg) and hypertension (>120/80 mmHg). The food habits of the participants had five options for frequency of consumption ranging from “never” to “daily.”

Vitamin D was classified as follows: <20 ng/ml (50 nmol/L) as “deficient” and between 21 and 29 ng/ml (50–75 nmol/L) as “insufficient” (19). The Homeostatic Model Assessment for Insulin Resistance (HOMA-IR) was calculated using the following formula: fasting insulin (microU/L) × fasting glucose (nmol/L)/22.5. Log(HOMA-IR) transforms the skewed distribution of fasting insulin values to assess a possible linear correlation with glucose clamp estimates of insulin sensitivity when extensive ranges of insulin sensitivity/resistance were being studied (20). GDM was classified based on the WHO guidelines: fasting blood sugar (FBS) ≥92 mg/dl and/or postprandial blood sugar (PPBS) ≥153 mg/dl. Anemia in pregnant women was categorized as follows: anemic for hemoglobin levels ≤11 g/dl, and not anemic for hemoglobin levels above 11 g/dl (21). Low birth weight was defined as weight at birth of <2,500 g. Preterm births were defined as those delivered below the gestational age of 37 weeks.

### Statistical analysis

Quantitative variables were described in terms of mean ± standard deviation (SD). Median and interquartile range (IQR) were used to describe a non-normal distribution and qualitative variables are reported as percentages. To compare the differences between the two Vitamin D status categories, the χ^2^ test (for nominal data), the one-way analysis of variance (ANOVA; for continuous variables with normal distribution), and the Mann–Whitney *U*-test (for continuous variables with non-normal distribution) were used. Spearman's correlation coefficient was used for non-normally distributed continuous variables. After preliminary examination of the data distribution, we log-transformed FBS, PPBS, Vitamin D, and Homeostatic Model Assessment of Insulin Resistance (HOMA-IR) values. Univariate and adjusted regression models were used to evaluate the associations between serum Vitamin D concentrations during pregnancy with GDM and low birth weight. Regression analysis models were adjusted for confounders for the association between LBW and Vitamin D levels.

In this cohort, 77% of mothers were Vitamin D deficient. Hence, quartiles were generated based on the Vitamin D level distribution to evaluate GDM at each level, with quartile 1 (<9.3) being the lowest and quartile 2 (<12.7) and quartile 3 (<18.5) being the highest. Quartile 4 (>18.6) serves as the reference group. The confounders were chosen based on *a priori* selected potential confounders. Age is included as a confounder as aging skin produces significantly lower amounts of Vitamin D compared to younger skin (22). Multiparity was associated with decreased Vitamin D levels (23). Religious factors like staying indoors and restrictive clothing too play an important factor in Vitamin D deficiency (24, 25), and studies have shown that socioeconomic status has a considerable impact on Vitamin D levels (26). Obesity is also proven to be a risk factor for Vitamin D deficiency (27). Model 1 was adjusted for sociodemographic factors such as maternal age, parity, religion, and socioeconomic status. Model 2 included factors in Model 1 in addition to biological factors such as maternal adiposity, while Model 3 included factors in Model 2 and season of blood sampling.

We conducted a causal mediation analysis to examine the indirect effect of the independent variable Vitamin D on the fasting and post-challenge glucose levels through mediator variables: insulin resistance, controlling for potential confounders that included maternal age, parity, skin-fold thickness, season, and socioeconomic status. The analysis was performed using the mediation package in R Studio 2022.07.2 (28, 29) based on the general approach to causal mediation analysis developed by Imai et al. (30). We estimated average causal mediation effects (ACME), average direct effects (ADE), and total effects, as well as the proportion of the total effect that is mediated. The mediation analysis consisted of two regression models: (1) a linear regression model predicting the mediators from Vitamin D levels and the confounders and (2) a linear regression model predicting the dependent fasting/post-challenge glucose levels, Vitamin D levels, and the confounders. To account for sampling variability and obtain robust estimates, we employed a bootstrap procedure with 1,000 simulations. Confidence intervals were estimated at the 95% level.

Linear regression was also carried out to check for associations between Vitamin D and glucose values after adjusting for potential confounders. Logistic regression analysis was conducted to check for the association between serum Vitamin D with LBW and GDM. Vitamin D was categorized based on percentiles and standard cut-off (20 ng/ml).

## Results

The sub-sample from the MAASTHI cohort comprised 230 pregnant women, and characteristics comparing those with Vitamin D values greater and <20 ng/ml are provided in Table 1. Among the participants, 77.3% were Vitamin D deficient, 19.1% were diagnosed with GDM, and 27.8% of the infants were born with low birth weight.

**Table 1:** Characteristics of pregnant women and their offspring and their association with Vitamin D levels (*N* = 230).

**Characteristics of the cohort**	**Categories**	**Total**	**Vitamin D deficiency**		***p*-value**
		***N*** **(%)/mean** ±**SD**	**Yes/**<**20 ng/ml (*****n*** = **178)**	**No/more than 20 ng/ml (*****n*** = **52)**	
Age (years)	Mean ± SD	24.2 ± 4.2	24.3 ± 4.1	23.8 ± 4.4	0.37
Gestational age at recruitment (weeks)	Mean ± SD	22.5 ± 4.9	22.9 ± 4.9	21.3 ± 4.5	**0.03**
Gestational age at the time of blood draw (weeks)	Mean ± SD	27.6 ± 2.2	27.6 ± 2.3	27.5 ± 2.1	0.66
Religion	Hinduism **(Ref)**	98 (42.6%)	64 (36.0)	34 (65.4%)	
	Christianity	9 (3.9%)	6 (3.4%)	3 (5.8%)	0.9
	Islam	123 (53.5%)	108 (60.7%)	15 (28.8%)	**0.00**
Participant education	Pre-university college or graduation **(Ref)**	76 (33.0)	59 (33.1)	17 (32.7)	
	Illiterate/primary/middle school	57 (24.8)	48 (27%)	9 (17.3)	0.34
	High school	97 (42.2)	71 (39.9)	26 (50.0)	0.50
Participant's occupation	Employed **(Ref)**	17 (7.4)	13 (7.3)	4 (7.7)	
	Unemployed	213 (92.6)	165 (92.7)	48 (92.3)	0.92
Socioeconomic class	Middle **(Ref)**	81 (35.2)	60 (33.7)	21 (40.4)	
	Lower	149 (64.8)	118 (66.3)	31 (59.6)	0.37
Parity	Nulliparous **(Ref)**	95 (41.3)	76 (42.7)	19 (36.5)	
	Primiparous	114 (49.6)	87 (48.9)	27 (51.9)	0.52
	Multiparous	21 (9.1)	15 (8.4)	6 (11.5)	0.39
Hemoglobin (g/dl)	Median (IQR)	11.1 (1.3)	11.1 (1.2)	11.2 (1.4)	0.80
Fasting blood glucose (mg/dl)	Median (IQR)	89 (9.3)	82 (10)	81 (8)	0.31
Postprandial blood glucose (mg/dl)	Median (IQR)	108 (30)	109 (31)	104.5 (24)	0.27
Insulin (pmol L)	Median (IQR)	46.6 (29.4)	48.27 (28.6)	38.73 (30.3)	**0.04**
HOMA-IR	Median (IQR)	1.5 (1.1)	1.6 (1.18)	1.3 (1.10)	**0.04**
Physical activity level in mothers (MET)	Median (IQR)	1,169 (342)	1,176.5 (327)	1,143 (379)	0.34
Season	Summer **(Ref)**	38 (16.5)	30 (16.9)	8 (15.4)	
	Rainy	141 (61.3)	104 (58.4)	37 (71.2)	0.51
	Post-monsoon	36 (15.7)	32 (18)	4 (7.7)	0.25
	Winter	15 (6.5)	12 (6.7)	3 (5.8)	0.93
Dairy consumption	Daily **(Ref)**	154 (67.0)	118 (66.3)	36 (69.2)	
	1–3 times per week	24 (10.4)	21 (11.8)	3 (5.8)	0.86
	Never	52 (22.6)	39 (21.9)	13 (25)	0.15
Fish consumption	1–3 times per week **(Ref)**	61 (26.5)	55 (30.9)	6 (11.5)	
	1–3 times per month	73 (31.7)	52 (29.2)	21 (40.4)	**0.01**
	Less than once per month	96 (41.7)	71 (39.9)	25 (48.1)	**0.00**
Calcium intake (g)	Median (IQR)	602.5 (304.8)	602.2 (306.4)	643 (284)	0.63
Sum of skin-fold in mother (mm)	Mean ± SD	47.1 ± 13.6	47.6 ± 13.7	45.2 ± 13.5	0.12
Hypertension (mmHg)	No **(Ref)**	215 (93.5)	165 (92.7)	50 (96.2)	
	Yes	15 (6.5)	13 (7.3)	2 (3.8)	0.38
GDM	No **(Ref)**	186 (80.9)	140 (78.7)	46 (88.5)	
	Yes	44 (19.1)	38 (21.3)	6 (11.5)	0.12
Preterm delivery	≥37 weeks **(Ref)**	214 (93.0)	166 (93.3)	48 (92.3)	
	<37 weeks	16 (7.0)	12 (6.7)	4 (7.7)	0.81
C-section	No **(Ref)**	126 (54.8)	97 (54.5)	29 (55.8)	
	Yes	104 (44.2)	81 (45.5)	23 (44.2)	0.87
Low birth weight (<2.5 Kg)	No **(Ref)**	166 (72.2)	134 (75.3)	32 (61.5)	
	Yes	64 (27.8)	44 (24.7)	20 (38.5)	0.05
Sum of skin-fold thickness in child (<10 percentile)	No **(Ref)**	203 (88.3)	160 (89.9)	43 (82.7)	
	Yes	27 (11.7)	18 (10.1)	9 (17.3)	0.16
Sum of skin-fold thickness in child (>90 percentile)	No **(Ref)**	206 (89.6)	158 (88.8)	48 (92.3)	
	Yes	24 (10.4)	20 (11.2)	4 (7.7)	0.46
Head circumference (<10 percentile)	No **(Ref)**	204 (88.7)	158 (88.8)	46 (88.5)	
	Yes	26 (11.3)	20 (11.2)	6 (11.5)	0.95
MUAC (<10 percentile)	No **(Ref)**	207 (90)	165 (92.7)	42 (82.8)	
	Yes	23 (10)	13 (7.3)	10 (19.2)	**0.01**

Bold values indicate *p* < 0.05.

The two groups had no statistically significant differences across various characteristics such as age, gestational age, education levels, socioeconomic class, parity, and season of test. We found that Islam religion and low fish intake were significantly associated with low Vitamin D levels. Vitamin D was also associated with maternal insulin, HOMA, low birth weight, and MUAC of the offspring. Low levels of Vitamin D were not associated with preterm delivery, c-section, and hypertension in mothers. The ANOVA test results suggest that there is no significant difference in mean Vitamin D levels across the four seasons [*F*_(3, 226)_ = 2.193, *p* = 0.090; Supplementary Tables 1, 2].

Vitamin D levels were associated with the HOMA levels in the mothers (β coefficient =-0.18, 95% CI −0.35, 0.01) even after adjusting for maternal age, parity, religion, socioeconomic status, and skin-fold thickness. Furthermore, for every one-unit increase in Vitamin D levels, HOMA-IR decreased by about 18%. These findings suggest that Vitamin D levels are associated with reduced insulin resistance in pregnant women. On the other hand, the associations between Vitamin D levels and fasting blood sugar and postprandial blood sugar were not statistically significant in the unadjusted and adjusted models (Table 2).

**Table 2:** Association between logarithmically transformed maternal Vitamin D status with logarithmically transformed glucose and insulin resistance levels in pregnant women.

**Variable**	**Unadjusted**			**Adjusted**		
	β **(SE)**	***p*** **-value**	**95% CI**	β **(SE)**	***p*** **-value**	**95% CI**
Log of fasting blood sugar (mg/dl)	−0.023 (0.01)	0.07	−0.49, 0.03	−0.02 (0.01)	0.07	−0.05, 0.00
Log of postprandial blood sugar (mg/dl)	−0.02 (0.28)	0.46	−0.07, 0.03	−0.02 (0.02)	0.44	−0.07, 0.39
Log HOMA (IR)	−0.20 (0.07)	**0.00**	−0.36, −0.05	−0.18 (0.08)	**0.03**	−0.35, 0.01

Adjusted for maternal age, parity, religion, socioeconomic status skin-fold thickness and season. Bold values indicate *p* < 0.05.

Table 3 presents the logistic regression estimates of the association between gestational diabetes (GDM) and Vitamin D levels during pregnancy using different models. The odds of GDM were 3.22 times higher for women in Quartile 1 (<9.3 ng/ml) after adjusting for age, parity, religion, socioeconomic status, skin-fold thickness, and season.

**Table 3:** Logistic regression estimates of the association of gestational diabetes for Vitamin D levels during pregnancy.

	**Quartile 1 (**<**9.3 ng/ml) (*****N*** = **58)**		**Quartile 2 (**<**12.7 ng/ml) (*****N*** = **58)**		**Quartile 3 (**<**18.5 ng/ml) (*****N*** = **57)**		**Quartile 4 (>18.6 ng/ml) *N* = 57**
	**OR (95% CI)**	***p*** **-value**	**OR (95% CI)**	***p*** **-value**	**OR (95% CI)**	***p*** **-value**	
GDM^a^	2.75 (0.97, 7.7)	**0.05**	2.26 (0.78, 6.50)	0.13	2.31 (0.80, 6.65)	0.12	1
Model 1	3.41 (1.1, 10.5)	**0.03**	2.83 (0.93, 8.65)	0.06	2.48 (0.82, 7.48)	0.10	1
Model 2	3.01 (1.02, 8.8)	**0.04**	2.371 (0.78, 7.19)	0.12	2.29 (0.76, 6.87)	0.14	1
Model 3	3.22 (1.03, 10.07)	**0.04**	2.25 (0.71, 7.14)	0.16	2.26 (0.74, 6.85)	0.15	1

^a^ = Unadjusted.

Model 1 = Adjusted for maternal age (years), parity, religion, and socioeconomic status.

Model 2 = Adjusted for maternal age (years), parity, religion, socioeconomic status, and maternal skin-fold thickness (mm).

Model 3 = Adjusted for maternal age (years), parity, religion, socioeconomic status, maternal skin-fold thickness (mm), and season. Bold values indicate *p* < 0.05.

We present the results of a mediation analysis exploring the role of insulin resistance (logarithmic value) in the relationship between log Vitamin D levels and log fasting and post-challenge glucose. The covariates include age, religion, parity, and socioeconomic status. While the direct effect of fasting and postprandial blood sugar on Vitamin D was not significant, the indirect effect through insulin resistance was statistically significant. A unit decrease in Vitamin D is associated with a decrease of 0.015 units in FBS as it flows through the mediator variable insulin resistance. Approximately 62% of the total effect of Vitamin D on FBS is explained by the mediator insulin resistance but is not statistically significant. A unit decrease in Vitamin D is associated with a decrease of 0.019 units in PPBS through the mediator variable insulin resistance. Approximately 51% of the total effect of Vitamin D on PPBS is explained by the mediator HOMA but is not statistically significant (Table 4).

**Table 4:** Mediation role of insulin resistance (HOMA) in the association of Vitamin D levels and GDM.

	**ACME**	**Direct effect**	**Total effect**	**Proportion mediated**	***p*-value for ACME**
Log of fasting blood glucose (mg/dl)	−0.015 (−0.03, 0.00)	−0.008 (−0.03, 0.02)	−0.024 (−0.05, 0.00)	0.62 (−0.76, 3.20)	0.02
Log of postprandial blood glucose (mg/dl)	−0.019 (−0.04, 0.00)	0.004 (−0.05, 0.05)	−0.02 (−0.07, 0.03)	0.51 (−5.15, 7.45)	0.02

ACME, Average Causal Mediation Effect.

We found that women who were Vitamin D deficient had twice the odds of giving birth to a low birth weight child (OR 2.04, CI 0.99, 4.19) as compared to women without Vitamin D deficiency. The association was significant even after adjusting for confounders (Table 5).

**Table 5:** Association between maternal vitamin D status with anthropometry of child.

**Exposure**	**Birth weight**						**MUAC**					
	**Unadjusted**			**Adjusted**			**Unadjusted**			**Adjusted**		
	β **(SE)/OR**	***p*** **-value**	**CI**	β **(SE)/OR**	***p*** **-value**	**CI**	β **(SE)/OR**	***p*** **-value**	**CI**	β **(SE)/OR**	***p*** **-value**	**CI**
Vitamin D (continuous)	−0.01	0.18	−0.02, 0.00	−0.04	0.49	**−0.0**11, 0.00	−0.08	0.18	−0.02, 0.00	−0.08	0.20	−0.02, 0.00
Log vitamin D	−0.05	0.37	−0.36, 0.13	−0.02	0.75	−0.29, 0.21	−0.08	0.22	−0.84, 0.19	−0.08	0.22	−0.88, 0.20
**Vitamin D (percentiles)**												
High (>75th percentile) (Ref)												
Moderate	1.55	0.21	0.78, 3.07	1.52	0.25	0.73, 3.15	0.34	**0.03**	0.12, 0.93	0.38	0.07	0.13, 1.11
Low	1.66	0.22	0.73, 3.74	1.60	0.29	0.66, 3.86	0.45	0.17	0.14, 1.4	0.40	0.16	0.11, 1.46
**Vitamin D (dichotomous)**												
More than 20 ng/ml (Ref)												
<20 ng/ml	1.90	0.05	0.98, 3.66	2.04	**0.05**	0.99, 4.19	0.33	**0.01**	0.13, 0.80	0.27	**0.01**	0.09, 0.74

Adjusted for age, religion, parity, maternal adiposity, and gestational age at delivery. Bold values indicate *p* < 0.05.

## Discussion

We report a high prevalence of Vitamin D deficiency among pregnant women attending public hospitals in Bengaluru city. Severe Vitamin D deficiency was associated with GDM and LBW in this sample.

The season of blood sampling did not demonstrate any association with Vitamin D deficiency among the pregnant women. Harinarayan et al. investigated how seasonal variations and the time of day affect the production of pre-vitamin D3 using an ampoule model in Tirupati, which is located in South India (latitude 13.40°N and longitude 77.2°E). This location corresponds closely to our study site in Bengaluru (latitude 12.98°N, longitude 77.5°E). Their findings revealed consistent synthesis of Vitamin D throughout the year without seasonal fluctuations. This consistency in Vitamin D synthesis across seasons could explain why we did not observe an association between season and Vitamin D deficiency in our study as well (31).

Notably, most samples in our cohort were collected during the rainy season, spanning from June to September. Despite this, our ANOVA analysis showed no significant difference in mean Vitamin D levels across seasons. Our results are consistent with a study conducted among pregnant women in Bengaluru that did not find any influence of season on Vitamin D levels (4). However, Marwaha et al. (32) reported in their study that the south zone (Pune and Hyderabad) showed a constant ultraviolet blood irradiation (UVBI) throughout the year, except in the months of July and August, where there was a slight dip probably due to cloudy and rainy weather. It is crucial to consider the notable differences in elevation between these locations and our study site in Bengaluru. Bengaluru is situated at an elevation almost double that of Pune and Hyderabad (+3,018 ft above the mean sea level compared to +1,840 ft and +1,778 ft, respectively), leading to significantly greater UV exposure at higher elevations compared to the sea level. These elevation-related differences may contribute to varying UVBI levels and could influence Vitamin D synthesis patterns, potentially explaining the lack of significant seasonal variation observed in our study.

In our study, we found that low consumption of fish was associated with Vitamin D deficiency, which was established by previous research as well (33). Apart from sun exposure and dietary habits, there are other genetic factors such as the altered activity of enzymes like 24-hydroxylase (CYP24A1) that can influence Vitamin D levels during pregnancy. CYP24A1 is capable of transforming 25(OH)D and 1,25(OH)2D to their inactive forms to protect target organs or tissues from excessive Vitamin D signaling.

Genetic mutants in the Vitamin D pathway (GC, CYP3A4, CYP24A1, and NADSYN1/DHCR7) have shown significant associations with 25(OH)D levels among pregnant women in southeast China (34). The crucial role of CYP24A1 has been proven by CYP24A1-knockout mice. CYP24A1-knockout mice manifested severe hypercalcemia, as well as ossification of bone due to the apparently low ability to catabolize 1,25(OH)2D (35). These genetic mutants could be associated with low levels of Vitamin D in the cohort, and this observation needs further genomic evaluation.

We found that pregnant women in the lowest quartile of Vitamin D levels have the highest odds of developing gestational diabetes compared to the reference group. In addition, Vitamin D levels were significantly associated with insulin resistance, and insulin resistance mediated a significant part of the association between Vitamin D and GDM.

Our study findings are consistent with previous metanalysis indicating the association between Vitamin D deficiency and the risk of GDM (36–39). The results from other cohort studies (40) and studies conducted within the country (4, 41) have revealed similar findings. However, there have also been studies with contradictory findings where Vitamin D hypovitaminosis was not associated with GDM (42, 43).

Accumulating evidence supports the fact that Vitamin D deficiency is associated with the pathogenesis of insulin resistance. Mechanistic evidence continues to demonstrate that Vitamin D plays a role in the metabolism of glucose and lipids in insulin-sensitive tissues like adipose tissue, skeletal muscle, and the liver (44). According to recent reviews, lack of Vitamin D may contribute to the molecular causes of insulin resistance (45). Vitamin D plays a crucial role in cellular processes responsible for glucose and lipid metabolism homeostasis via the insulin signaling pathway. Vitamin D participates in insulin secretion by pancreatic β-cells through the regulation of intracellular Ca^2+^ concentration (46). Disturbances in insulin signaling and inflammation are closely related, and Vitamin D was found to reduce both of these disorders (46). Low maternal Vitamin D levels continue to exert a lasting impact on infants. Krishnaveni et al. showed that low 25(OH)D concentrations during pregnancy were associated with higher insulin resistance in 9-year-old children. The results from several meta-analyses offer support for the fact that supplementation of Vitamin D could ameliorate the condition of GDM and reduce adverse maternal–neonatal outcomes (47–49). It is imperative to conduct well-designed intervention trials to determine whether Vitamin D administration will enhance glycemic control in women with GDM in this sub-population. We also showed that pregnant women with low Vitamin D deficiency (<20 ng/ml) have a greater risk of giving birth to low-birth-weight babies. The relationship between maternal Vitamin D and LBW is complex and might be influenced by various factors, including genetics, diet, sun exposure, and supplementation. The role of Vitamin D in regulating calcium balance and its influence on parathyroid hormone levels within the body can have a substantial impact on the growth of the fetus. A meta-analysis of 16 studies by Fang et al. (50) established that maternal Vitamin D deficiency had an increased risk of low birth weight (OR = 2.39; 95% CI 1.25–4.57; *p* = 0.008). One of the reported mechanisms is the impact of inadequate maternal Vitamin D levels on calcium absorption and bone metabolism, which could lead to a decrease in the accumulation of fetal bone mass (51). Furthermore, a notable correlation was observed between placental weight and the area under the curve (AUC) of [25(OH)D], which reflects the maternal Vitamin D levels throughout pregnancy, as well as the birth weight of the newborn (52).

The results of this study have important implications for pregnant women and healthcare providers. Lower Vitamin D levels (<9.5 ng/ml) during pregnancy were associated with an increased risk of gestational diabetes and low birth weight of babies. This finding is similar to the study in Bengaluru that found that the lowest quartile (≤23.6 nmol/L or 9.4 ng/ml) of Vitamin D concentration had a significantly higher proportion of GDM compared to those in the higher quartiles of Vitamin D (4) In light of these findings, it is imperative to examine if the current deficiency thresholds for Vitamin D, commonly used in clinical practice, are appropriate for Indian populations. There is a need for re-evaluation of deficiency thresholds, taking into account the specific risk profiles and health outcomes associated with different levels of deficiency within the Indian context.

This finding also emphasizes the importance of maintaining optimal Vitamin D levels, especially among individuals at the risk of severe deficiency. Public health interventions should focus on strategies to address severe deficiency, such as appropriate supplementation, dietary changes, and increased sunlight exposure.

The role of insulin resistance in mediating the association between Vitamin D levels and gestational diabetes suggests that interventions that target insulin resistance can be implemented. In pregnancy, lifestyle changes and other interventions that target insulin resistance and Vitamin D deficiency may be effective in improving glucose control in women with GDM. Future research needs to focus on developing interventions useful in the local settings. For example, recommendations are made to undertake physical exercises three times per week in the sunshine to improve glycemic control in GDM patients and the reduced incidence of GDM in pregnant women with obesity (53).

The strengths of this study include the measurement of maternal Vitamin D status and insulin levels during the critical period; our study provides important insights into the association between these factors and gestational diabetes among women visiting government hospitals for antenatal care. In addition, we showed using mediation analysis that insulin resistance is a key mediator in the development of gestational diabetes. We did a comparison between the subsample selected for this analysis and the entire MAASTHI cohort; the subsample adequately represented the larger cohort in terms of key demographic or clinical characteristics that could influence the associations studied (Supplementary Table 3). However, the limitations include the lack of analysis regarding determinants of Vitamin D deficiency, such as sunlight exposure duration and the absence of pre-pregnancy body mass index (BMI) values, which are important confounders in the relationship between Vitamin D levels, insulin resistance, gestational diabetes, and birth weight.

In conclusion, our study adds to the growing body of evidence that Vitamin D plays an important role in glucose metabolism during pregnancy and low birth weight. Furthermore, we show the role of insulin resistance in the association between maternal Vitamin D deficiency and GDM in South Indian pregnant women. These results could form the basis for developing more comprehensive public health strategies and policies concerning this matter. Gallo et al., in their meta-analysis, provide evidence that Vitamin D supplementation significantly decreases maternal HOMA-IR and increases infant birth weight (54). Further research is needed to explore the precise mechanisms underlying this association and to determine the optimal strategies for monitoring and managing Vitamin D levels during pregnancy.

## Data availability statement

The raw data supporting the conclusions of this article will be made available by the authors, without undue reservation.

## Ethics statement

The studies involving humans were approved by Institutional Ethics Committee of Indian Institute of Public Health- Bangalore. The studies were conducted in accordance with the local legislation and institutional requirements. The participants provided their written informed consent to participate in this study.

## Author contributions

RD: Conceptualization, Formal analysis, Investigation, Methodology, Project administration, Validation, Writing – original draft. OS: Supervision, Writing – review & editing. GB: Conceptualization, Formal analysis, Funding acquisition, Writing – review & editing.

## References

[1] Jiang Z Pu R Li N Chen C Li J Dai W. High prevalence of vitamin D deficiency in Asia: a systematic review and meta-analysis. Crit Rev Food Sci Nutr. (2023) 63:3602–11. 10.1080/10408398.2021.1990850 34783278

[2] Jeyakumar A Shinde V Ravindran R. Pooled estimate of vitamin D deficiency among pregnant women in India: a systematic review and meta-analysis. J Health Popul Nutr. (2021) 40:28. 10.1186/s41043-021-00253-y 34187594 PMC8243731

[3] Sheela Ravinder S Padmavathi R Maheshkumar K Mohankumar M Maruthy KN Sankar S. Prevalence of vitamin D deficiency among South Indian pregnant women. J Family Med Prim Care. (2022) 11:2884–9. 10.4103/jfmpc.jfmpc_1819_21 36119194 PMC9480695

[4] Dwarkanath P Vinotha P Thomas T Joseph S Thomas A Shirley G. Relationship of early vitamin D concentrations and gestational diabetes mellitus in Indian pregnant women. Front Nutr. (2019) 6:116. 10.3389/fnut.2019.00116 31448279 PMC6691186

[5] Shin JS Choi MY Longtine MS Nelson DM. Vitamin D effects on pregnancy and the placenta. Placenta. (2010) 31:1027–34. 10.1016/j.placenta.2010.08.015 20863562 PMC2993775

[6] Dave A Verma M Jain N Dave A. A study of vitamin D levels and associated deficiency in pregnancy and its effect on maternal and fetal outcome. Int J Reprod Contracept Obstet Gynecol. (2016) 6:84–8. 10.18203/2320-1770.ijrcog20164637

[7] Zhang M-X Pan G-T Guo J-F Li B-Y Qin L-Q Zhang Z-L. Vitamin D deficiency increases the risk of gestational diabetes mellitus: a meta-analysis of observational studies. Nutrients. (2015) 7:8366–75. 10.3390/nu7105398 26437429 PMC4632418

[8] Akbari S Khodadadi B Ahmadi SAY Abbaszadeh S Shahsavar F. Association of vitamin D level and vitamin D deficiency with risk of preeclampsia: a systematic review and updated meta-analysis. Taiwan J Obstet Gynecol. (2018) 57:241–7. 10.1016/j.tjog.2018.02.013 29673668

[9] Bodnar LM Platt RW Simhan HN. Early-pregnancy vitamin D deficiency and risk of preterm birth subtypes. Obstet Gynecol. (2015) 125:439. 10.1097/AOG.0000000000000621 25569002 PMC4304969

[10] McFarland MB Trylovich CG Langer O. Anthropometric differences in macrosomic infants of diabetic and nondiabetic mothers. J Matern Fetal Med. (1998) 7:292–5. 10.1002/(SICI)1520-6661(199811/12)7:6<292::AID-MFM7>3.0.CO;2-A 9848695

[11] Walsh JM McGowan CA Kilbane M McKenna MJ McAuliffe FM. The relationship between maternal and fetal vitamin d, insulin resistance, and fetal growth. Reprod Sci. (2013) 20:536–41. 10.1177/1933719112459222 22968764 PMC3713539

[12] Wimalawansa SJ. Associations of vitamin D with insulin resistance, obesity, type 2 diabetes, and metabolic syndrome. J Steroid Biochem Mol Biol. (2018) 175:177–89. 10.1016/j.jsbmb.2016.09.017 27662816

[13] Dutta D Maisnam I Shrivastava A Sinha A Ghosh S Mukhopadhyay P. Serum vitamin-D predicts insulin resistance in individuals with prediabetes. Indian J Med Res. (2013) 138:853–60. 24521626 PMC3978972

[14] Kayaniyil S Vieth R Retnakaran R Knight JA Qi Y Gerstein HC. Association of vitamin D with insulin resistance and β-cell dysfunction in subjects at risk for type 2 diabetes. Diabetes Care. (2010) 33:1379–81. 10.2337/dc09-2321 20215450 PMC2875459

[15] Chen X Chu C Doebis C von Baehr V Hocher BJ. Sex-dependent association of vitamin D with insulin resistance in humans. J Clin Endocrinol Metab. (2021) 106:e3739–47. 10.1210/clinem/dgab213 34406392

[16] Chen YH Liu ZB Ma L Zhang ZC Fu L Yu Z. Gestational vitamin D deficiency causes placental insufficiency and fetal intrauterine growth restriction partially through inducing placental inflammation. J Steroid Biochem Mol Biol. (2020) 203:105733. 10.1016/j.jsbmb.2020.105733 32784046

[17] Wang X Jiao X Tian Y Zhang J Zhang Y Li J. Associations between maternal vitamin D status during three trimesters and cord blood 25(OH)D concentrations in newborns: a prospective Shanghai birth cohort study. Eur J Nutr. (2021) 60:3473–83. 10.1007/s00394-021-02528-w 33661376

[18] Kumar BR Dudala SR Rao A. Kuppuswamy's socio-economic status scale–a revision of economic parameter for 2012. Int J Res Dev Health. (2013) 1:2–4.

[19] F Holick MJ. Vitamin D: evolutionary, physiological and health perspectives. Curr Drug Targets. (2011) 12:4–18. 10.2174/138945011793591635 20795941

[20] Duncan MH Singh BM Wise PH Carter G Alaghband-Zadeh J. A simple measure of insulin resistance. Lancet. (1995) 346:120–1. 10.1016/S0140-6736(95)92143-5 7603193

[21] World Health Organization. Haemoglobin Concentrations for the Diagnosis of Anaemia and Assessment of Severity. Geneva: World Health Organization (2011).

[22] Lips P. Vitamin D physiology. Prog Biophys Mol Biol. (2006) 92:4–8. 10.1016/j.pbiomolbio.2006.02.016 16563471

[23] Andersen LB Abrahamsen B Dalgård C Kyhl HB Beck-Nielsen S Frost-Nielsen M. Parity and tanned white skin as novel predictors of vitamin D status in early pregnancy: a population-based cohort study. Clin Endocrinol. (2013) 79:333–41. 10.1111/cen.12147 23305099

[24] Suchitra M Shanthi T Balachandar S Parthasarathy S. Estimating serum vitamin D levels and assessing its influencing factors among antenatal women in a South Indian town-Kumbakonam urban rural epidemiological study: KURES-5. Ind J Med Spec. (2021) 12:22. 10.4103/injms.injms_107_20

[25] Hatun S Islam Om Cizmecioglu F Kara B Babaoglu K Berk F. Subclinical vitamin D deficiency is increased in adolescent girls who wear concealing clothing. J Nutr. (2005) 135:218–22. 10.1093/jn/135.2.218 15671216

[26] Sharma S Kumar A Prasad S Sharma S. Current scenario of vitamin D status during pregnancy in north Indian population. J Obstet Gynaecol India. (2016) 66:93–100. 10.1007/s13224-014-0658-5 27046962 PMC4818828

[27] Bodnar LM Catov JM Roberts JM Simhan HN. Prepregnancy obesity predicts poor vitamin D status in mothers and their neonates. J Nutr. (2007) 137:2437–42. 10.1093/jn/137.11.2437 17951482 PMC2556251

[28] Tingley D Yamamoto T Hirose K Keele L Imai K. Mediation: R package for causal mediation analysis. J. Stat Softw. (2014) 59:1–38. 10.18637/jss.v059.i0526917999

[29] Hicks R Tingley D. Causal mediation analysis. Stata J. (2011) 11:605–19. 10.1177/1536867X1101100407

[30] Imai K Keele L Tingley D. A general approach to causal mediation analysis. Psychol Methods. (2010) 15:309. 10.1037/a0020761 20954780

[31] Harinarayan CV Holick MF Prasad UV Vani PS Himabindu G. Vitamin D status and sun exposure in India. Dermatoendocrinol. (2013) 5:130–41. 10.4161/derm.23873 24494046 PMC3897581

[32] Marwaha RK Yenamandra VK Sreenivas V Sahay R Baruah MP Desai A. Regional and seasonal variations in ultraviolet B irradiation and vitamin D synthesis in India. Osteoporos Int. (2016) 27:1611–7. 10.1007/s00198-015-3427-0 26630977

[33] Wu BT Dyer RA King DJ Innis SM. Low fish intake is associated with low blood concentrations of vitamin D, choline and n-3 DHA in pregnant women. Br J Nutr. (2013) 109:936–43. 10.1017/S0007114512002103 22691303

[34] Shao B Jiang S Muyiduli X Wang S Mo M Li M. Vitamin D pathway gene polymorphisms influenced vitamin D level among pregnant women. Clin Nutr. (2018) 37:2230–7. 10.1016/j.clnu.2017.10.024 29153269

[35] Masuda S Byford V Arabian A Sakai Y Demay MB St-Arnaud R. Altered pharmacokinetics of 1α, 25-dihydroxyvitamin D3 and 25-hydroxyvitamin D3 in the blood and tissues of the 25-hydroxyvitamin D-24-hydroxylase (Cyp24a1) null mouse. Endocrinology. (2005) 146:825–34. 10.1210/en.2004-1116 15498883

[36] Poel YHM Hummel P Lips P Stam F van der Ploeg T Simsek S. Vitamin D and gestational diabetes: a systematic review and meta-analysis. Eur J Intern Med. (2012) 23:465–9. 10.1016/j.ejim.2012.01.007 22726378

[37] Lu M Xu Y Lv L Zhang M. Association between vitamin D status and the risk of gestational diabetes mellitus: a meta-analysis. Arch Gynecol Obstet. (2016) 293:959–66. 10.1007/s00404-016-4010-4 26825733

[38] Milajerdi A Abbasi F Mousavi SM Esmaillzadeh A. Maternal vitamin D status and risk of gestational diabetes mellitus: a systematic review and meta-analysis of prospective cohort studies. Clin Nutr. (2021) 40:2576–86. 10.1016/j.clnu.2021.03.037 33933723

[39] Amraei M Mohamadpour S Sayehmiri K Mousavi SF Shirzadpour E Moayeri A. Effects of vitamin D deficiency on incidence risk of gestational diabetes mellitus: a systematic review and meta-analysis. Front Endocrinol. (2018) 9:7. 10.3389/fendo.2018.00007 29449829 PMC5800395

[40] Mosavat M Arabiat D Smyth A Newnham J Whitehead L. Second-trimester maternal serum vitamin D and pregnancy outcome: the Western Australian Raine cohort study. Diabetes Res Clin Pract. (2021) 175:108779. 10.1016/j.diabres.2021.108779 33766698

[41] Muthukrishnan J Dhruv G. Vitamin D status and gestational diabetes mellitus. Indian J Endocrinol Metab. (2015) 19:616–9. 10.4103/2230-8210.163175 26425469 PMC4566340

[42] Farrant HJW Krishnaveni GV Hill JC Boucher BJ Fisher DJ Noonan K. Vitamin D insufficiency is common in Indian mothers but is not associated with gestational diabetes or variation in newborn size. Eur J Clin Nutr. (2009) 63:646–52. 10.1038/ejcn.2008.14 18285809 PMC2678985

[43] Gashlan HM Noureldeen AF Elsherif HA Tareq O. Vitamin D and insulin resistance in gestational diabetes mellitus. J Diabetes Endocrinol. (2017) 8:17–25. 10.5897/JDE2017.011138147025

[44] Narvaez CJ Simmons K Brunton J Salinero A Chittur S Welsh J. Induction of STEAP 4 correlates with 1, 25-dihydroxyvitamin D3 stimulation of adipogenesis in mesenchymal progenitor cells derived from human adipose tissue. J Cell Physiol. (2013) 228:2024–36. 10.1002/jcp.24371 23553608

[45] Zhou Q Wen S Liu M Zhang S Jin X Liu AJ. Association between gene polymorphisms of vitamin D receptor and gestational diabetes mellitus: a systematic review and meta-analysis. Int J Environ Res Public Health. (2021) 18:205. 10.3390/ijerph18010205 33383970 PMC7794905

[46] Doyle ME Egan JM. Pharmacological agents that directly modulate insulin secretion. Pharmacol Rev. (2003) 55:105–31. 10.1124/pr.55.1.7 12615955

[47] Wang M Chen Z Hu Y Wang Y Wu Y Lian F. The effects of vitamin D supplementation on glycemic control and maternal-neonatal outcomes in women with established gestational diabetes mellitus: a systematic review and meta-analysis. Clin Nutr. (2021) 40:3148–57. 10.1016/j.clnu.2020.12.016 33386179

[48] Ojo O Weldon SM Thompson T Vargo E. The effect of vitamin D supplementation on glycaemic control in women with gestational diabetes mellitus: a systematic review and meta-analysis of randomised controlled trials. Int J Environ Res Public Health. (2019) 16:1716. 10.3390/ijerph16101716 31100793 PMC6572053

[49] Zhang Y Gong Y Xue H Xiong J Cheng G. Vitamin D and gestational diabetes mellitus: a systematic review based on data free of Hawthorne effect. BJOG. (2018) 125:784–93. 10.1111/1471-0528.15060 29244241

[50] Fang K He Y Mu M Liu K. Maternal vitamin D deficiency during pregnancy and low birth weight: a systematic review and meta-analysis. J Matern Fetal Neonatal Med. (2021) 34:1167–73. 10.1080/14767058.2019.1623780 31122092

[51] Ioannou C Javaid M Mahon P Yaqub M Harvey N Godfrey K. The effect of maternal vitamin D concentration on fetal bone. J Clin Endocrinol Metab. (2012) 97:E2070–7. 10.1210/jc.2012-2538 22990090 PMC3485609

[52] Mead MJ McWhorter CA Rodgers MD Ebeling MD Shary JR Gregoski MJ. Does maternal vitamin D status influence placental weight or vascular and inflammatory pathology? Secondary analysis from the Kellogg Pregnancy Study. J Steroid Biochem Mol Biol. (2023) 233:106358. 10.1016/j.jsbmb.2023.106358 37414103 PMC11229515

[53] Colberg SR Sigal RJ Yardley JE Riddell MC Dunstan DW Dempsey PC. Physical activity/exercise and diabetes: a position statement of the American Diabetes Association. Diabetes Care. (2016) 39:2065–79. 10.2337/dc16-1728 27926890 PMC6908414

[54] Gallo S McDermid JM Al-Nimr RI Hakeem R Moreschi JM Pari-Keener M. Vitamin D supplementation during pregnancy: an evidence analysis center systematic review and meta-analysis. J Acad Nutr Diet. (2020) 120:898–924.e4. 10.1016/j.jand.2019.07.002 31669079

