# Supplementary material for: Low levels of Vitamin D during pregnancy associated with gestational diabetes mellitus and low birth weight: results from the MAASTHI birth cohort
Source: Front Nutr. 2024 Jun 3;11:1352617. doi: 10.3389/fnut.2024.1352617 (PMC11180835; doi:10.3389/fnut.2024.1352617)
Supplement: Supplementary file 1 [file Table_1.docx]

Supplementary Table 1: Mean Vitamin D levels across different seasons

|  | N | Mean | Std. Deviation | Std. Error |
| --- | --- | --- | --- | --- |
|  |  |  |  |  |
| Summer | 38 | 14.4 | 7.1 | 1.15 |
| Post monsoon | 36 | 12.0 | 6.1 | 1.02 |
| Rainy | 141 | 15.4 | 7.5 | 0.63 |
| Winter | 15 | 13.2 | 7.5 | 1.94 |
| Total | 230 | 14.5 | 7.3 | 0.48 |

Supplementary Table 2: ANOVA results of Vitamin D levels across different seasons

|  | Sum of Squares | df | Mean Square | F | Sig. |
| --- | --- | --- | --- | --- | --- |
| Between Groups | 349.131 | 3 | 116.377 | 2.193 | .090 |
| Within Groups | 11992.355 | 226 | 53.064 |  |  |
| Total | 12341.486 | 229 |  |  |  |

Supplementary Table 3: Demographic and Clinical parameters between main cohort and the subsample studied in this cohort.

|  | **Main cohort**  **(N=2962)** | **Subsample**  **(N=230)** |
| --- | --- | --- |
| **Age** (Mean ± SD) | 24.2 ±4.06 | 24.2 ± 4.2 |
| **Religion** |  |  |
| Hinduism | 42.6% | 50.5% |
| Christianity | 3.9% | 5.5% |
| Islam | 53.5% | 44.1% |
| **Parity** |  |  |
| Nulliparous | 45.6% | 41.3% |
| Multiparous | 54.4% | 58.7% |
| **Socioeconomic status** |  |  |
| Lower | 54.6% | 64.8% |
| Middle | 44.8% | 35.2% |
| **Participant’s Occupation** |  |  |
| Employed | 7.4% | 7.1% |
| Unemployed | 92.6% | 92.9% |
| **Participant’s Education** |  |  |
| College/ Graduation | 34.2% | 33.0 |
| Illiterate/Primary/  Middle School | 22.1% | 24.8 |
| High School | 43.7% | 42.2 |
| **Skinfold thickness (in mm)** (Mean ± SD) | 46.54±10.05 | 47.1±13.6 |
